# Persistence of Anticancer Activity in Berry Extracts after Simulated Gastrointestinal Digestion and Colonic Fermentation

**DOI:** 10.1371/journal.pone.0049740

**Published:** 2012-11-21

**Authors:** Emma M. Brown, Gordon J. McDougall, Derek Stewart, Gema Pereira-Caro, Rocio González-Barrio, Philip Allsopp, Pamela Magee, Alan Crozier, Ian Rowland, Chris I. R. Gill

**Affiliations:** 1 Northern Ireland Centre for Food and Health, Centre for Molecular Biosciences, University of Ulster, Coleraine, N. Ireland, United Kingdom; 2 Environmental and Biochemical Sciences Group, Enhancing Crop Productivity and Utilization Theme, The James Hutton Institute, Dundee, United Kingdom; 3 Plant Products and Human Nutrition Group, School of Medicine, University of Glasgow, Glasgow, United Kingdom; 4 Hugh Sinclair Unit of Human Nutrition, Department of Food and Nutritional Sciences, University of Reading, Reading, United Kingdom; 5 School of Life Sciences, Heriot Watt University, Edinburgh, United Kingdom; National Cancer Center, Japan

## Abstract

Fruit and vegetable consumption is associated at the population level with a protective effect against colorectal cancer. Phenolic compounds, especially abundant in berries, are of interest due to their putative anticancer activity. After consumption, however, phenolic compounds are subject to digestive conditions within the gastrointestinal tract that alter their structures and potentially their function. However, the majority of phenolic compounds are not efficiently absorbed in the small intestine and a substantial portion pass into the colon. We characterized berry extracts (raspberries, strawberries, blackcurrants) produced by *in vitro-*simulated upper intestinal tract digestion and subsequent fecal fermentation. These extracts and selected individual colonic metabolites were then evaluated for their putative anticancer activities using *in vitro* models of colorectal cancer, representing the key stages of initiation, promotion and invasion. Over a physiologically-relevant dose range (0–50 µg/ml gallic acid equivalents), the digested and fermented extracts demonstrated significant anti-genotoxic, anti-mutagenic and anti-invasive activity on colonocytes. This work indicates that phenolic compounds from berries undergo considerable structural modifications during their passage through the gastrointestinal tract but their breakdown products and metabolites retain biological activity and can modulate cellular processes associated with colon cancer.

## Introduction

The etiology of colorectal cancer, the fourth most common cause of cancer related mortality globally, has strong associations with diet [1], [2]. Given the inverse correlation of fruit and vegetable consumption with colorectal cancer incidence it is unsurprising that bioactive phytochemicals within these foods are of interest with regard to their anticancer properties [2], [3]. Phenolic compounds are abundant in soft fruit such as berries, and include anthocyanins, flavonols, flavan-3-ols, ellagitannins, proanthocyanidins, hydroxycinnamates and phenolic acids [4]. These isolated phytochemicals or whole berry extracts have been reported to exert putative anticancer effects in colonocytes both *in vitro* and *in vivo* [3], [5], [6]. For example, commercial bilberry extracts decreased proliferation of HT29 adenocarcinoma cells *in vitro* [7], in tumor cells in F344 rats treated with azoxymethane [8] and in tumor cells from biopsies in colorectal cancer patients [9].

Berry phenolic compounds typically occur as glycosides with, for example, anthocyanins comprising a diversity of conjugates including glucosides, galactosides, rutinosides, xylorutinosides and sambubiosides. Such conjugated glycosides are, for the main part, not absorbed in the small intestine but those with a glucose moiety may be subject to deconjugation, catalysed by membrane-bound lactase phlorizin hydrolase in the intestinal epithelium and/or soluble β-glucosidases, producing aglycones which are more lipophilic and therefore absorbed by passive diffusion [10], [11]. However, the majority of phenolic compounds are not efficiently absorbed in the small intestine [4] and a substantial portion pass into the colon [12]. Once in the large intestine, these components are subject to degradation by the colonic microflora forming catabolites including simple phenolic and aromatic acids [10], [13]. Therefore, it is reasonable to infer that the colonic epithelium is in direct contact with both the parent phenolic compounds and their degradation products [14].

Generally, studies on putative anticancer activity have used *in vitro* colonocyte cell models with extracts rich in phenolic compounds or purified components from berries and have not considered the effects of their *in vivo* metabolites. The initial aim of this study was to produce and characterize extracts derived from raspberries (*Rubus idaeus L*.) strawberries (*Fragaria*x *ananassa* ) and blackcurrants (*Ribes nigrum L*.) that were more representative of the components to which the colonic epithelium is exposed *in vivo*. This involved a combination of *in vitro* digestion and subsequent fecal fermentation of berry extracts to mimic the physiological changes encountered during passage through the gastrointestinal tract. The resultant extracts were then evaluated for biological activity using *in vitro* models representing key stages in colorectal cancer, namely, initiation, promotion and invasion.

## Materials and Methods

### Reagents

DMEM and fetal bovine serum (FBS) were obtained from Gibco Life Technologies Ltd (Paisley, Scotland, UK). All other chemicals were purchased from Sigma-Aldrich Company Ltd (Dorset, England, UK) unless otherwise specified.

### Berry extracts

Raspberries (*Rubus idaeus L*. cv. Glen Ample) and strawberries (*Fragaria*x *ananassa* cv. Elsanta) were obtained from local farmers, around Dundee, UK, while blackcurrants (*Ribes nigrum L*. cultivar 8982-6) were acquired from Bradenham Hall, Norfolk, UK. No specific permits were required for the described field studies as these berries were part of a larger crop grown by a farmer under contract to the James Hutton Institute and therefore not a field study, no permissions were required. The location was not protected in any way and no endangered or protected species were involved. Phenolic-rich extracts of the berries were obtained using the method of McDougall *et al*. [15]. *In vitro* digestion of the berries was modified from the method by Gil-Izquierdo *et al*. [16] as described previously [15]. Briefly, extract was obtained from the different berry species by homogenising berries with an equal volume of solvent (2% (v/v) glacial acetic acid in acetonitrile) in a Waring blender (full power, 3×15s), the homogenate filtered through muslin, and the filtrate subjected to rotary evaporation to remove the solvent. This extract then underwent an *in vitro* procedure that simulates the digestive process. Two sequential steps were performed; an initial pepsin/HCl step to simulate gastric conditions, and bile salts/pancreatin digestion to simulate conditions of the small intestine. This ‘colon-available’ extract underwent solid phase extraction to remove phenolics from bile salts present in the samples. Total anthocyanins were measured using a pH differential technique. The extract was then diluted in distilled water to a concentration of 500 µg gallic acid equivalents (GAE) as measured using the Folin-Ciocalteau method and dried in a speed-vac. Berry extract was stored at −20°C until use.

### In vitro fermentation

The *in vitro* digested (IVD) berry extracts were subjected to *in vitro* fermentation with human fecal samples to produce a sample representative of what may be present in the colon [17] Basal medium was prepared to sustain the fecal inoculum (in 500 mL of water: peptone water [2 g], yeast extract [2 g], NaCl [0.1 g], K_2_HPO_4_ [0.04 g], KH_2_PO_4_ [0.04 g], MgSO_4_.7H_2_O [0.01 g], CaCl_2_.6H_2_O [0.01 g], NaHCO_3_ [2 g], Tween 89 [2 mL], hemin [0.05 g], vitamin K (10 µl), L-cysteine HCl [0.5 g]). A further 1500 mL of water was added with stirring. Once all components had dissolved completely the medium was dispensed in 135 mL aliquots into glass bottles and autoclaved.

The fermentation vessels were maintained at 37°C, sterile medium added and nitrogen gas was pumped through the vessels to maintain anaerobic conditions. The pH was maintained at ∼6.6 using the inserted pH electrodes and addition of acid and base solutions (0.5 M HCl/0.5 M NaOH respectively).

IVD berry extract (10 mL) was added to the basal medium in separate fermentation vessels and a control vessel constructed without berry extract. Fecal samples were collected with the prior approval of the ethics committee of the University of Reading. This experiment was carried out using fecal samples from three different volunteers. After obtaining verbal informed consent, a standard questionnaire to collect information regarding the health status, drugs use, clinical anamnesis, and lifestyle was administrated before the donor was asked to provide a fecal sample. The University of Reading ethics committee exempted this study from review because no donors were involved in any intervention and waived the need for written consent due to the fact the samples received were not collected by means of intervention. Fresh fecal samples were provided by 3 apparently healthy individuals (2 males, 1 female, and age range 24–37 years, who had not taken antibiotics within the previous 3 months). Within 2 hours of collection, the samples were combined and 10% (w/v) fecal slurry prepared using sterile PBS. Fecal slurry, 15 mL, was added to each of the fermentation vessels and allowed to mix thoroughly with the medium for 5 min and the pH adjusted accordingly. At time T = 0 h, 6 mL of fecal slurry/basal medium was removed from each vessel, ultracentrifuged for 2×10 min at 10,000 *g* and the supernatant (termed fermented berry extract) filtered through 0.22 µm filters before being aliquoted into Eppendorf tubes. Sampling was repeated at T = 24 hours and all aliquots stored at −80°C.

### Liquid chromatography-mass spectroscopy

Berry extracts corresponding to 40 µg of gallic acid equivalents (GAE) were analyzed on a LCQ-DECA system comprised of Surveyor autosampler, pump, a photodiode array detector and a Thermo-Finnigan ion trap mass spectrometer as outlined by Coates *et al*. [18]. Phenolic components were identified on the basis of a combination of co-chromatography with reference compounds, retention times, and absorbance and mass spectra (Table S1). Classes of polyphenols were quantified against relevant standards: hydroxycinnamates as 5-*O*-caffeoylquinic acid equivalents; flavonols as quercetin-*3-O*-glucoside equivalents, anthocyanins as cyanidin-3-*O*-glucoside equivalents; ellagitannins and ellagic acids as ellagic acid equivalents and others as catechin equivalents. Parent compounds in the berry IVD extracts were analysed by LC-MS as described by Borges *et al*. [19] and phenolic acids in fermentates by GC-MS according to procedures of Roowi *et al*. [20].

### Berry extracts and individual phenolics

Bermudez-Soto *et al*. [21] estimated that consumption of approximately 640 mg of phenolic compounds would result in colonic concentrations in the region of 160 mg/l. The dose range in this study equated maximally to 50 mg/l and as such is physiologically relevant. For experimentation, the IVD extracts were reconstituted in growth medium to a concentration of 500 µg/mL GAE, and subsequently diluted to produce the dose range of 50, 25, 12.5, 6.25, and 3.125 µg/mL GAE. During experimentation cells were pre-incubated with each concentration of berry extract for 24 hours at 37°C, 5% CO_2_, 95% humidity. Fermented berry extracts were diluted 1/10 v/v with PBS prior to testing. Individual phenolic acids were used at a similar concentrations to those phenolic acids detected in the fermented berry extracts by LC-MS analysis (Table 1), a common range was selected for comparison purposes namely, 1 and 5 or 5 and 10 µg/mL depending on the concentration of the individual phenolic acid in the fermented extract. Twenty four hours was selected as the exposure time for all *in vitro* studies as it is generally considered to reflect the average colonic transit time, after pre-incubation with treatments the cells were washed and harvested with trypsin prior to use in the various assays.

**Table 1:** Quantities of phenolic acids in fermented berry extracts.

Compound	Control	Raspberry	Strawberry	Blackcurrant
Benzoic acid	2.4±0.5	2.3±0.4	2.3±0.0	1.7±0.1
4-Hydroxybenzoic acid	n.d.	0.26±0.01*	0.52±0.07*	0.25±0*
3,4-Dihydroxybenzoic acid	n.d.	n.d.	n.d.	0.49±0.0*
Tyrosol	0.15±0.0	0.32±0.0*	0.32±0.07*	0.17±0.0
Phenylacetic acid	16±4	34±4*	21±1.4	7.4±0.1
4′-Hydroxyphenylacetic acid	1.5±0.2	3.7±0.5*	3.0±0.3*	5.3±0.3*
3-(Phenyl)propionic acid	1.0±0.3	2.1±0.3	3.6±0.2*	20.7±1*
3-(4′-Hydroxyphenyl)propionic acid	n.d.	n.d.	n.d.	1.7±0.2*

a Data expressed as mean values in μg/mL ± standard deviation. (*) indicates values significantly higher than Control figures at *p*<0.05; n.d, not detected.

### Tissue culture

Given that the colonic epithelium maybe in direct contact with both parent phenolic compounds and their degradation products [14] the bioactivity of berry extracts was assessed using colonocyte cell-based assays that represent early and late stage events in carcinogenesis, namely initiation [22], [23] and invasion [24], [25]. Cell lines were used as models for colorectal cancer, namely HT29 human colorectal adenocarcinoma cell line for anti-genotoxicity/genotoxicity, mutagenicity studies and HT115 a human colon carcinoma cell line for matrigel invasion studies. The HT29, HT115 and MRC5 human lung fibroblast-like cells were obtained from the European Collection of Cell Cultures, Salisbury, UK and were cultured as described by Coates *et al*. [18]. HT29 (G17 neo) cells were a gift from Professor Bill Kauffman, University of North Carolina, USA and were maintained in DMEM supplemented with 10% FBS.

### Biological activity assays

#### Cytotoxicity assay

Cytotoxicity of the berry extracts on HT29 cells treated for 24 hours was determined using the MTT (3-(4,5-dimethylthiazol-2-yl)-2,5-diphenyltetrazolium bromide) colorimetric assay to assess cell viability in accordance with the method described by Coates *et al*. [18]. The experiment was repeated independently three times for each treatment and the mean results expressed as % cell survival normalized to biological control.

#### Genotoxicity assay (Comet assay)

The effect of the IVD berry extracts and fermented berry extract on colonocyte DNA damage was determined using the Comet assay according to the method described previously [18]. HT29 cells were pre-incubated with the various berry extracts for 24 h. The anti-genotoxic potential of the extract was assessed by treating pre-incubated HT29 cells with hydrogen peroxide (75 µM H_2_O_2_). To determine any genotoxic effects of the berry extract, pre-incubated cells were treated with PBS instead of H_2_O_2_. Each experiment was performed independently 3 times and presented as mean % tail DNA normalised to control.

#### Matrigel invasion and migration assays

Invasion of tumour cells through the basement membrane is an important step in the metastasis process. Invasion itself involves multiple stages including degradation of the extracellular matrix, adhesion to and migration through the basement membrane. The invasion assay was conducted as described previously [18]. Briefly, HT115 cells were incubated with medium containing the various extracts tested for 24 h prior to experimentation and then seeded on to Matrigel™ (ECM matrix) coated Boyden chambers (or to an uncoated Boyden chambers when the migration assay was conducted) and allowed to invade for 24 h in response to chemo-attractant factor secreted by MRC 5 cells grown in the basolateral compartment of the boyden chamber. Growth medium was removed from the insert and cells were fixed in 70% v/v ethanol for 30 min prior to staining with haematoxylin. The number of invasive (on basolateral membrane surface) and non-invasive (on apical membrane surface) cells were counted in 5 random fields of view at x 20 magnification using the Kromascan Meteor 2 software (Kinetic Imaging Ltd, Liverpool, UK), and the percentage invasion calculated. Each experiment was repeated independently 3 times for each treatment and expressed as % invasion normalised to control.

#### Mutation frequency assay

This assay used HT29 (G17 neo) cells, which are HT29 cells (ATCC Cell Biology Collection, USA) transfected with a construct of 17 G-bases inside the coding sequence for the *neo* gene, which codes for resistance to the antibiotic neomycin. This insertion deactivates the gene and the cells become susceptible to neomycin, which can be reversed if a frameshift mutation occurs. The antibiotic geneticin (G418 sulfate), an analogue of neomycin, prevents cells that lack resistance from replicating, and thus from forming colonies. Fecal water, the aqueous phase of the fecal stream, has been shown to cause a 2–3 fold increase in frameshift mutations in HT29 cells (unpublished data). In this assay, fecal water is used as a biological control to induce a frameshift mutation, reverting cells to G418 resistance. The acridine mutagen ICR 191 induces frameshift mutations specifically at guanine bases and was used in this assay as a positive control [26]. The protective effect of berry extracts against fecal water-induced mutations was assessed. Fecal water was prepared as described by Boyd *et al*. [27] from a single male donor (age 32 years) after written informed consent had been obtained and prior to provision of the stool sample, with the approval of the University of Ulster ethical committee.

HT29 (G17 neo) cells were seeded into 6-well tissue culture plates at 1.5×10^4^ cells/well and incubated for 24 h at 37°C with 5% CO_2_ and 95% humidity. Growth medium was removed from all wells, IVD berry extracts and fermented berry extracts added to the test plates; growth medium was replenished in the control plate then incubated for 24 h. Medium was removed from all wells and the following treatments were added. Serum-free growth medium was used as a negative control, ICR 191 at 1 µg/mL in serum-free growth medium and fecal water (100 µL in 900 µL serum-free growth medium) were used as positive controls. Fecal water (100 µL in 900 µL serum-free growth medium) was added to each well in the test plate, and both plates were incubated for 1 h at 37°C with 5% CO_2_ and 95% humidity. Fecal water and controls were removed and the cells harvested using 0.25% trypsin-EDTA. Cells were disaggregated by needle aspiration in growth medium and counted using a Vi-CELL counter (Beckman-Coulter Ltd, High Wycombe, Buckinghamshire, United Kingdom). Cells were seeded into Petri dishes (1000 cells/dish) and 8 mL growth medium was added. Four dishes per treatment were seeded to be grown with/without G418 in duplicate. Cells were incubated for 24 h and G418 (80 µL of 100 mg/mL) was added to half the plates. The medium was refreshed every 3 days (replacing G418 each time). Whereas cells grown without G418 were stained after 7–10 days, cells grown with G418 were grown for up to 3 weeks. Cells were washed with PBS and fixed in 100% methanol for 5 min, the methanol removed and left to dry. The cells were stained with Giemsa stain (1∶20 in dH_2_O) for 15 min. The stain was gently rinsed off and the dishes were left to dry overnight. Colonies were counted and the mutation frequency was calculated for each plate using: Colony forming efficiency (CFE), mutation frequency (MF) and relative mutation frequency (RMF):
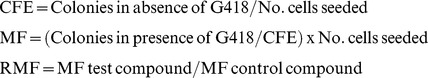



Each experiment was performed in duplicate for each treatment (2× G418+/2× G418−), and repeated three times independently and the results expressed as mean RMF.

### Statistical analysis

The mean of each data set was used for statistical analysis. The Shapiro-Wilk test was used to test for normality. Analysis of variance was applied to test for significant differences between means using ANOVA, Dunnett T and T3 tests. Four-way comparisons were performed between berry types to test for significance using Bonferroni. Significance was accepted at *p*<0.05. Analysis was carried out using SPSS (version 17.0 for Windows).

## Results

### In vitro digestion of berry extracts

The levels of individual phenolic compounds in the berry extracts pre- and post-IVD samples were analysed by LC-MS and the data obtained is summarized in Tables S1, S2, and S3 and Figure S1. The most abundant components in raspberry extracts were the ellagitannins, sanguiin H-10 and lambertianin C and the anthocyanin cyanidin-3-*O*-sophoroside. The strawberry extracts contained mainly anthocyanins and ellagitannins with smaller amounts of hydroxycinnamate derivatives and flavonols, whereas blackcurrant was dominated by four main anthocyanins which made up ∼70% of the total phenol content.

The recovery of phenolic components after IVD followed patterns which have been outlined previously [18]. Anthocyanins were generally less stable and the total anthocyanin content decreased in all three berry extracts post-IVD. However, there was no clear relationship between either the structure of the anthocyanidin aglycone or the glycosyl group and the stability of the anthocyanin to IVD. The stability of individual components also differed between berry extracts (e.g. the recovery of pelargonidin-3-*O*-glucoside was 52% in strawberry but 29% in raspberry) and stability appears to be dependent on the overall phenolic composition of each extract as suggested previously [15].

Overall, the pattern of recovery of ellagitannins suggested breakdown. Ellagitannins in strawberry and raspberry showed differential stability with larger components (e.g. lambertianin C and sanguiin H10 in raspberry) being reduced in content whereas the levels of smaller components (sanguiin H6) were either increased or maintained. This most likely reflects the well-documented breakdown of larger ellagitannins in slightly alkaline conditions through loss of ellagic acid after lactonization of a hexahydroxydiphenoyl group [28] yielding smaller ellagitannins [29]. Flavonol components were generally more stable to IVD and actually increased in relative yield presumably due to reductions in other components. Hydroxycinnamate derivatives were also more stable to IVD with e.g. a feruloyl-*O*-hexoside in strawberry increasing substantially.

### In vitro fermentation of berry extracts

LC-MS analysis established that phenolic compounds present at the start of the fermentation were absent after a 24 h fecal incubation while the levels of simpler aromatic components increased in the fermented extracts (Table 1). The main catabolites that increased significantly in most of the extracts were phenylacetic acid, 3-(phenyl)propionic acid, 4-hydroxybenzoic acid and 4′-hydroxyphenylacetic acid. There were some suggestions of berry-specific products as, for example, 3, 4-dihydroxybenzoic acid was only identified in the fermentates from black currant.

### Cytotoxicity

**Figure 1:**
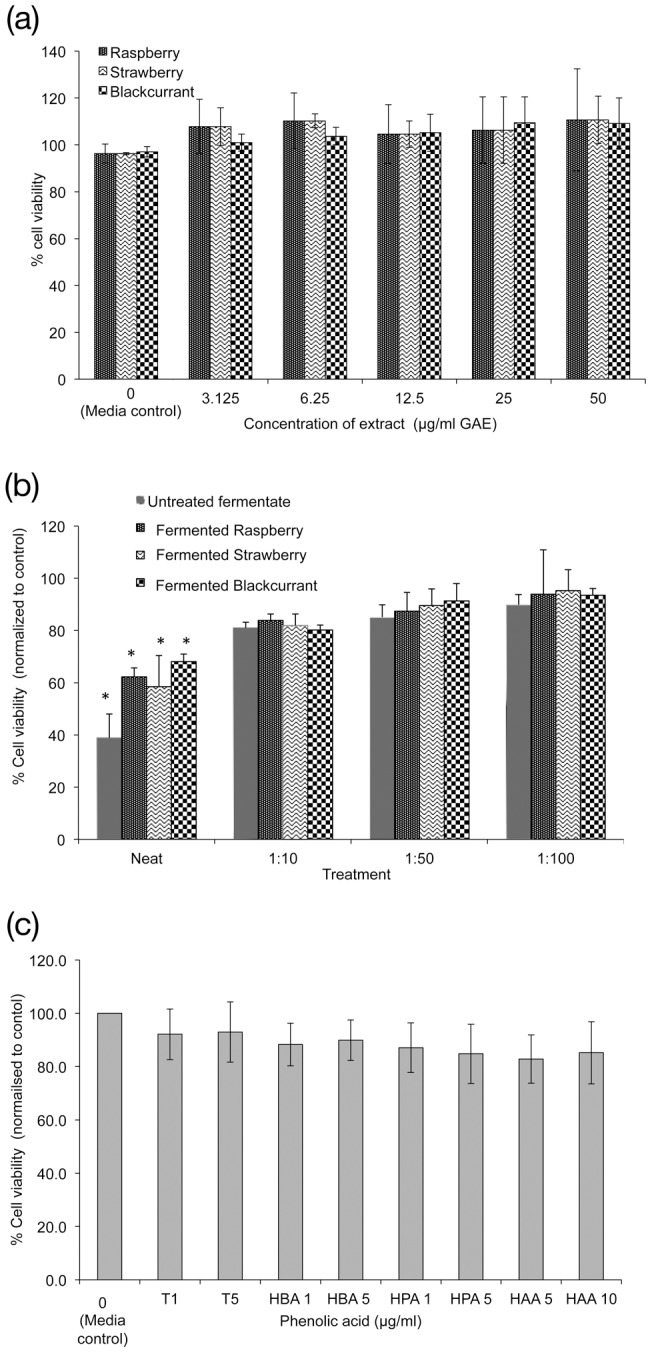
The cytotoxic effects of various types of berry extracts on HT29 cells. (A) Effects of IVD berry extract, (B) fermented berry extract at different dilutions in growth medium, (C) individual polyphenols- tyrosol (T), 3-(3′-hydroxyphenyl)propionic acid (HPA), 4-hydroxybenzoic acid (HBA), 4′-hydroxyphenylacetic acid (HAA) at 1, 5 or 10 µg/mL concentrations. Data presented is mean of 3 independent experiments + SD. One-way ANOVA and Dunnett T test, * *p*<0.05, significance is compared to media control (0 ug/mL) for A & C and against control fermentate in B. Phenol levels for 1∶10 dilution of fermented extract were raspberry 15.5, strawberry 13.9 and blackcurrant 12.4 µg/mL GAE.

**Figure 2:**
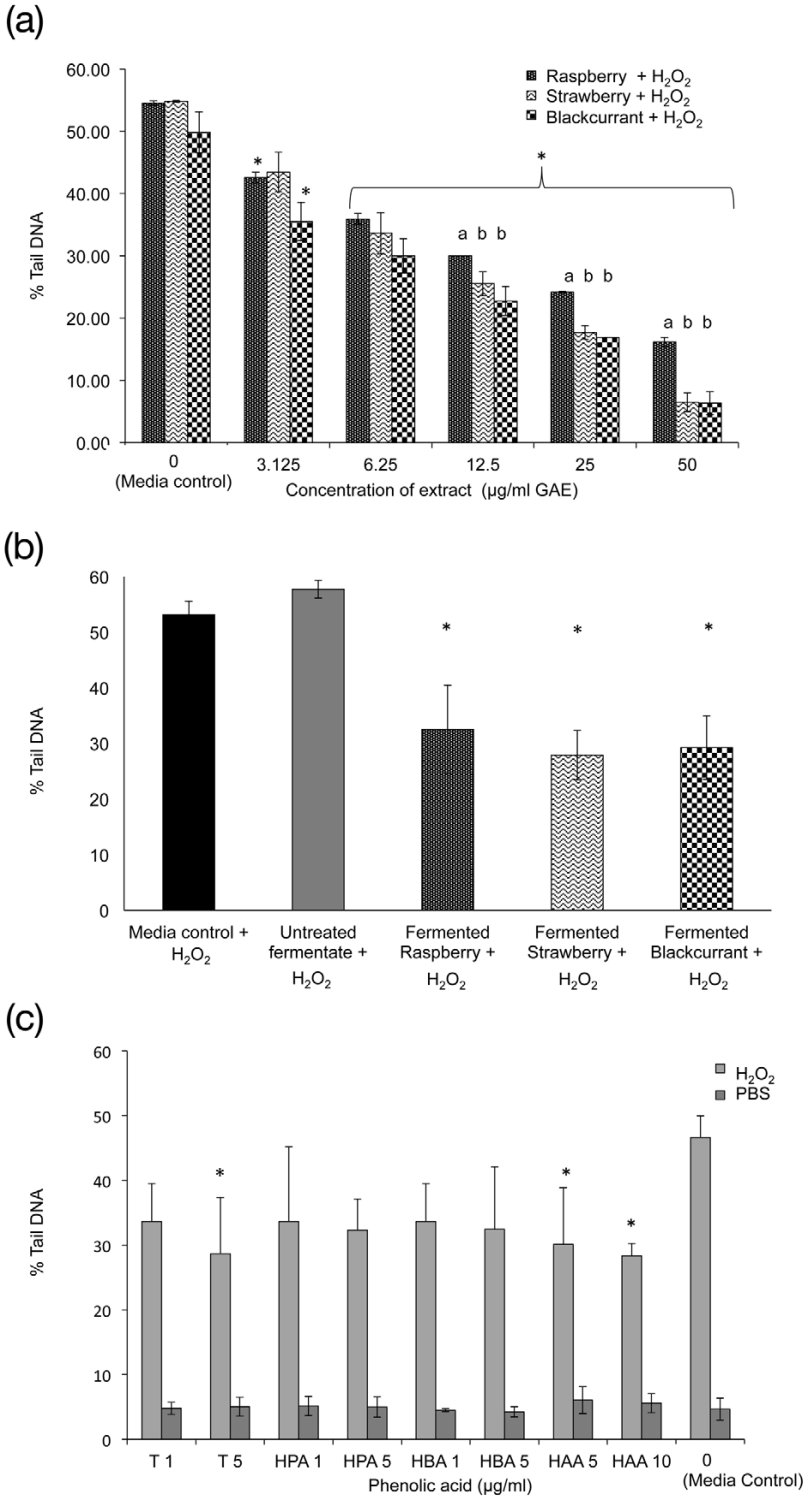
The anti-genotoxic effects of various types of berry extracts on HT29 cells. (A) Effects of IVD berry extract, (B) berry fermentates at a 1∶10 dilution after 24 hr pre-incubation on DNA damage in HT29 cells challenged with 75 µM H_2_O_2_ (phenol levels for berry fermentates were raspberry 15.5, strawberry 13.9, blackcurrant 12.4 µg/mL GAE), (C) individual polyphenols – tyrosol (T), 3-(3′-hydroxyphenyl)propionic acid (HPA), 4-hydroxybenzoic acid (HBA), 4′-hydroxyphenylacetic acid (HAA) at 1, 5 or 10 µg/mL concentrations. Data is presented as mean of 3 independent experiments + SD. One-way ANOVA and Dunnett T test, * p<0.05, significance is compared to media control (0 µg/mL) for A & C and against control fermentate in B. Bonferroni 4-way comparison, bars with different letters are significantly different from each other *p*<0.05.

No cytotoxic activity was observed for any of the IVD berry extracts (0–50 µg/mL GAE) compared to media control (at 1.6±0.1 AU) (Figure 1A). Further no anti-proliferative effects were observed as determined by growth curve and cell cycle analysis in accordance with methods described by McCann *et al*. [30]. All the fermented berry extracts were cytotoxic when used neat (reconstituted in 1.5 mL of growth medium) but notably were 25–50% less cytotoxic than the control fermentate (at 1.1±0.1 AU). A 1∶10 dilution was selected for all experiments as it represented the lowest dilution tested at which berry fermentates and control fermentate had no significant difference in cytotoxicity (Figure 1B). The individual phenolics identified in IVD fermentate exerted no cytotoxicity when tested at physiologically relevant concentrations compared to the media control (Figure 2C).

### Genotoxicity (Comet assay)

When measured by the Comet assay all IVD berry extracts above 6.25 µg/mL GAE exerted significant dose dependent anti-genotoxic effects on H_2_O_2_–challenged HT29 cells with no evidence of genotoxicity when compared to the media control. Overall, strawberry and blackcurrant IVD extracts reduced DNA damage more effectively than raspberry (*p*<0.01) (Figure 2A). Raspberry extract at maximal concentration decreased the % tail DNA from around 50% in H_2_O_2_–challenged HT29 cells to about 15%, whereas strawberry and blackcurrant treatment reduced levels to approximately half that value (7% tail DNA).

**Figure 3:**
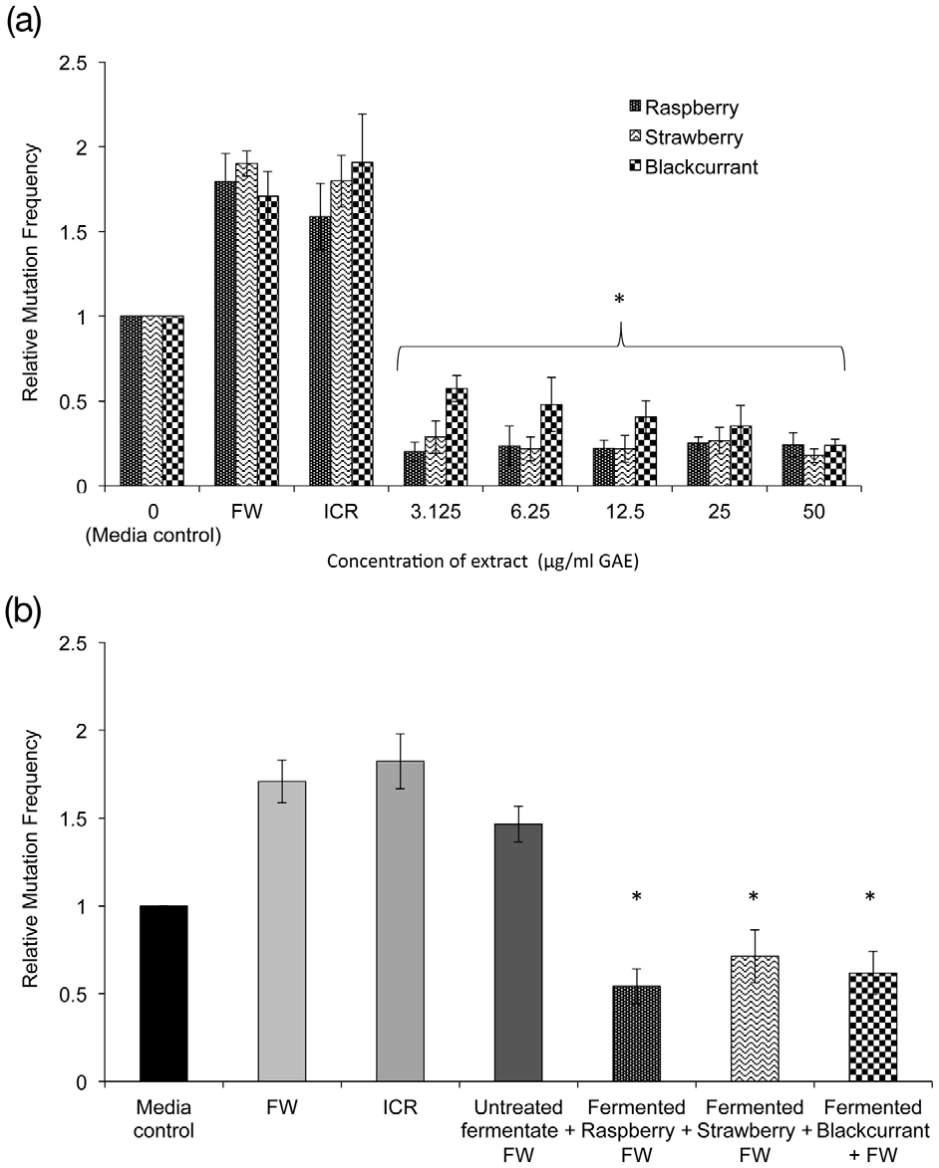
The anti-mutagenic effects of various types of berry extracts against fecal water-induced frameshift mutations in HT29 (G17 neo) cells. (A) Effects of IVD berry extracts, (B) berry fermentates. Data presented as mean relative mutation frequency of 3 independent experiments + SD. One-way ANOVA and Dunnett T test, * p<0.05, significance is compared to fecal water control for A and against control fermentate FW in B. Phenol levels for berry fermentates were raspberry 15.5, strawberry 13.9 and blackcurrant 12.4 µg/mL GAE.

All berry fermentates exhibited significant anti-genotoxic activity (*p*<0.001) decreasing % tail DNA to ∼30% (Figure 2B) compared to the control fermentate with no berry extract. The total phenol levels for the berry fermentates ranged from 12–16 µg/mL GAE and the observed reduction in tail DNA was comparable in magnitude to that reported for IVD berry extracts at 12.5 µg GAE/mL. Figure 3C indicates that the individual phenolic compounds tested had no genotoxicity activity and decreased H_2_O_2_ – induced DNA damage by ∼30% but only tyrosol (5 µg/mL) and 4′-hydroxyphenylacetic acid (5 and 10 µg/mL), reached significance (P>0.05).

### Mutation frequency assay

IVD berry extracts and fermented berry extracts did not display any mutagenic activity. ICR 191 and fecal water challenge both induced frameshift mutations in HT29 (G17 neo) resulting in an increase in relative mutation frequency (RMF) of ∼80%. Given the biological relevance of fecal water [31], it was selected in preference to the chemical mutagen ICR 191 as the challenge agent for use in the study.

All IVD berry extracts exhibited significant anti-mutagenic activity (>50% decrease in RMF) at all concentrations tested compared to fecal water (Figure 3A), but there was no significant difference between the efficacy of the individual IVD berry extracts. The berry fermentates also induced significant reductions in RMF post-fecal water challenge, compared to untreated fermentate (Figure 3B). A maximum reduction in RMF of 46% was observed with the fermented raspberry extract (RMF  = 0.54±0.10) and, as observed for the IVD extracts, there was no significant difference in the efficacy of different fermentates.

### Matrigel invasion assay and migration assay

**Figure 4:**
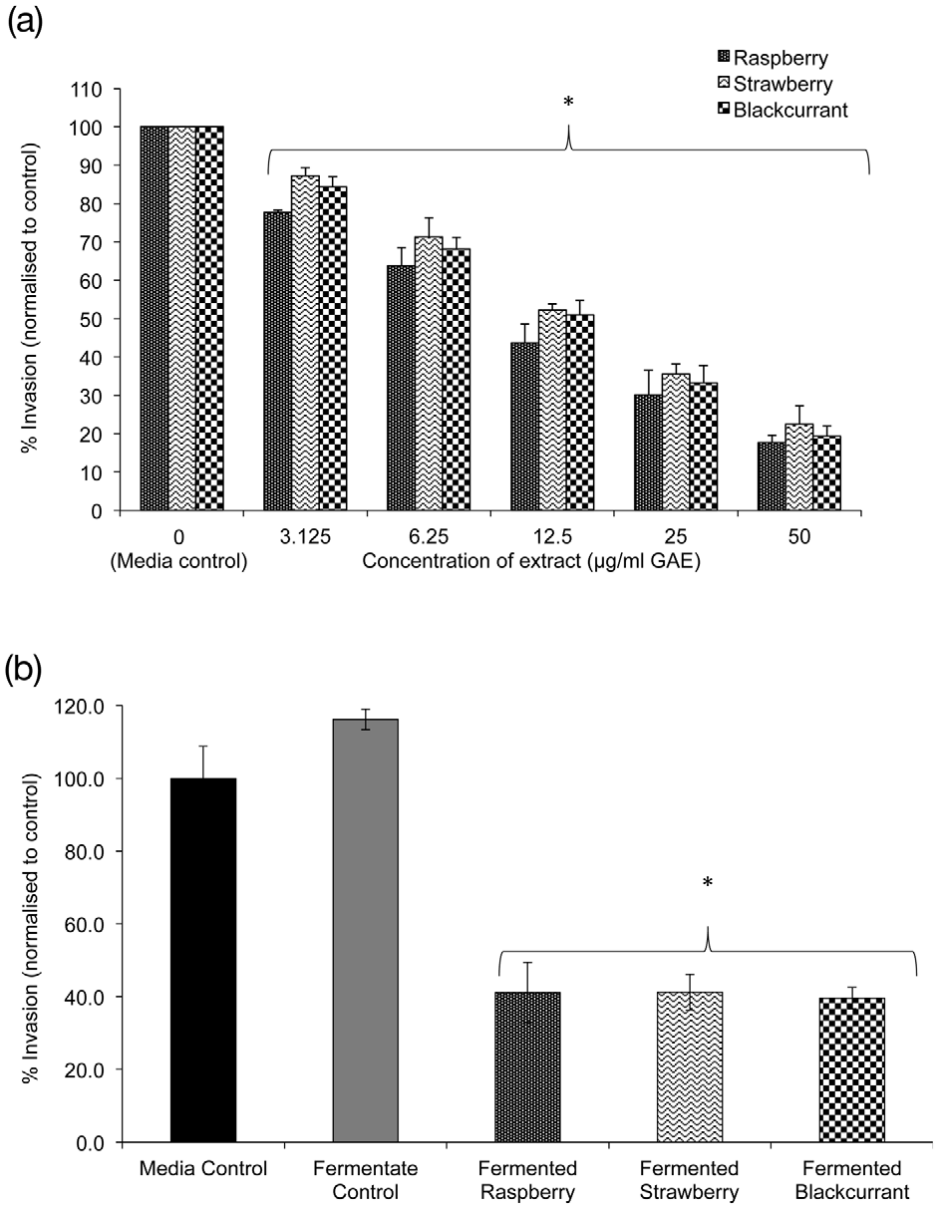
The anti-invasive effects of various types of berry extracts on HT115 cells. (A) Effects of IVD berry extracts, (B) berry fermentates. Mean of 3 independent experiments + SD, One-way ANOVA and Dunnett T test, * p<0.05, significance is compared to media control (0 µg/mL) for A and against control fermentate in B. Values are expressed as % cell invasion normalised to control. Berry extracts had no effect on migration of HT115 cells (data not shown). Phenol levels for berry fermentates were raspberry 15.5, strawberry 13.9, blackcurrant 12.4 µg/mL GAE.

Twenty four hour pre-incubation with IVD berry extracts inhibited invasion of HT115 cells in the matrigel invasion assay (Figure 4A). A significant dose-dependent anti-invasive effect was observed for blackcurrant, strawberry and raspberry (3.125–50 µg/mL GAE, P<0.05) when compared to the media control. All invasion data for IVD berry extracts was normalized to the invasion rate of the relevant media control (raspberry 25.7%, strawberry 22.1%, blackcurrant 19.3%) and were comparable to previously reported values [18]. Similarly all berry fermentates (Figure 4B) exhibited significant (P<0.01) anti-invasive effects reducing invasion to 40% of their respective untreated control (27%). Again there were no significant differences in the magnitude of effect observed between the berry fermentates. Furthermore, it is interesting to note that the reduction in the invasion of HT115 cells for both the IVD berry extracts and berry fermentates occurred with no change to the basal level of migration for the HT115 cells (data not shown) indicating that the anti-invasive effect was unlikely to be related to cell motility, but rather adhesion or membrane degradation.

## Discussion

Phenolic compounds in the diet undergo extensive modification by mammalian enzymes and the gut microbiota during their transit through the human body [32]. The implication of such metabolism on the bioactivity of berry polyphenols has not been systematically investigated. Consequently we subjected berry extracts to *in vitro* digestive and fermentative processes and used a physiologically-relevant dose range with a 24 hour exposure to test bioactivity using *in vitro* models representing key stages in colon carcinogenesis. Where significant effects were observed for the IVD extracts, the correspondent berry fermentates also exhibited chemoprotective activity, as did some of the individual phenolic compounds. Furthermore, digested and fermented extracts of equivalent phenolic content exerted an effect of similar magnitude in the bioassays, suggesting that products generated from *in vitro* digestion and fermentation of whole berries showed similar chemoprotective properties.

The three berry extracts used in this study contained distinct and characteristic phenolic profiles. After IVD, certain components were depleted. In general, anthocyanins were depleted during the IVD process (Table S1, Figure S1) and this is in agreement with previous studies on pomegranate, raspberry, chokeberry and blackberry juices [15], [21], [33], [34]. The effect of *in vitro* digestion on flavonols is less well studied: generally they were more stable during the digestive process with the recoveries of flavonols in strawberry and blackberry extracts either increasing or remaining constant (Table S2). Recoveries of the ellagitannins, sanguiin H-6, sanguiin H-10, and lambertinian C were similar to those previously reported [18]. Nevertheless, the recoveries of phenolic compounds after IVD were dependent on the type and composition of the individual berry extract and no general model for phenolic stability after IVD can be proposed. This makes it all the more relevant that digestion studies are carried out.

It is clear that both the digestive and fermentation processes altered the polyphenol composition relative to the original extract and thus reinforces the importance of testing physiologically-relevant polyphenol breakdown products rather than isolated parent compounds. The phenolic metabolites derived from colonic fermentation of berry components may accumulate to sufficient levels to play important roles in the colon but can be reabsorbed into general circulation and affect other processes [35]. For example, the urolithins being microbiota-derived catabolites of ellagitannins and ellagic acid are more effective than the parent compounds with respect to inhibition of the Wnt signaling pathway [36] and Nf-κB mediated anti-inflammatory activity [37]. It is, therefore, of interest to note that both the digested and fermented berry extracts were effective in protecting against DNA damage and frame-shift mutations caused by exogenous challenge agents indicating that activity was retained after bacterial fermentation. Furthermore, individual pure phenolic compounds representing bacterial fermentation catabolites, namely tyrosol and 4′-hydroxyphenylacetic acid, exhibited significant anti-genotoxic activity. Similar to the trends observed for reduction of DNA damage, all the digested and fermented berry extracts exerted a significant (P>0.05) anti-mutagenic activity against a relevant biological challenge (fecal water) [31], again demonstrating the preservation of bioactivity following digestion and fermentation. While anti-mutagenic activity has been reported for some phenolics [38] and fruit/vegetable extracts [39], [40] none considered the effects of digestion or fermentation on bioactivity.

Beyond activity related to the inhibition of the initiation of carcinogenesis, namely DNA damage and mutation, the berry phytochemicals also exhibited activity related to metastasis. In the present study, IVD berry extracts reduced invasiveness in a dose dependent manner similar to our previously reported data for an IVD raspberry extract [18]. Again, the berry extracts retained their activity following fermentation and significantly inhibited invasion of HT115 cells. Migration of HT115 cells was unaffected by the addition of any of the berry extracts which suggests that the berry extracts may act upon surface adhesion or enzymatic degradation rather than on cell on motility. While a few studies have shown that fruit extracts including mulberry have decreased invasion in a range of cell lines including HT29 [41], HCT116 [42], A549 [43] and MDA-MB-231 [44] they did not consider the role of digestion and fermentation on the bioactivity of the extracts. Similar consideration should also be given to activity related to cell proliferation as various berry extracts exhibit anti-proliferative activity in a range of colonocyte cell lines [45]. While it is currently beyond the scope of this paper, it would no doubt be of interest to investigate the impact of digestion and fermentation on such effects.

In conclusion, it would appear that berry extracts retain their biological activity after digestion and fermentation despite considerable structural modification and this strongly suggests that breakdown products and individual colonic metabolites, including, tyrosol and 4′–hydroxyphenylacetic acid, can modulate cellular processes associated with colon cancer. One can envisage that depending on their position in the colon, epithelial cells are likely to be exposed to varying concentrations of a mixture of phenolic components that have survived digestion in the upper gastrointestinal tract and their fermentation products and this study suggests that both sets of phenolic components can beneficially influence events relevant to the development of colon cancer.

## Supporting Information

Figure S1 **LC-MS traces for raspberry and blackcurrant extracts (A) and IVD extracts (B).** For each panel the top trace represents the scan at 520 nm. The masses for each peak are given along with the full scale deflection value. Peak labels correspond with the putative identities in Table S1.
(DOCX)

Table S1 **Total phenol and anthocyanin content of berry extracts pre-simulated digestion, IVD and post SPE, n = 8.**
(DOCX)

Table S2 **Putative identities and quantities of (poly)phenolic compounds detected in berry extracts before and after IVD^a^.**
(DOCX)

Table S3 **Putative identities of peaks detected in pre-digested berry extracts.**
(DOCX)
