# Supplementary material for: Persistence of Anticancer Activity in Berry Extracts after Simulated Gastrointestinal Digestion and Colonic Fermentation
Source: PLoS One. 2012 Nov 21;7(11):e49740. doi: 10.1371/journal.pone.0049740 (PMC3504104; doi:10.1371/journal.pone.0049740)
Supplement: Figure S1 — LC-MS traces for raspberry and blackcurrant extracts (A) and IVD extracts (B). For each panel the top trace represents the scan at 520 nm. The masses for each peak are given along with the full scale deflection value. Peak labels correspond with the putative identities in Table S1. (DOCX) [file pone.0049740.s001.docx]

**
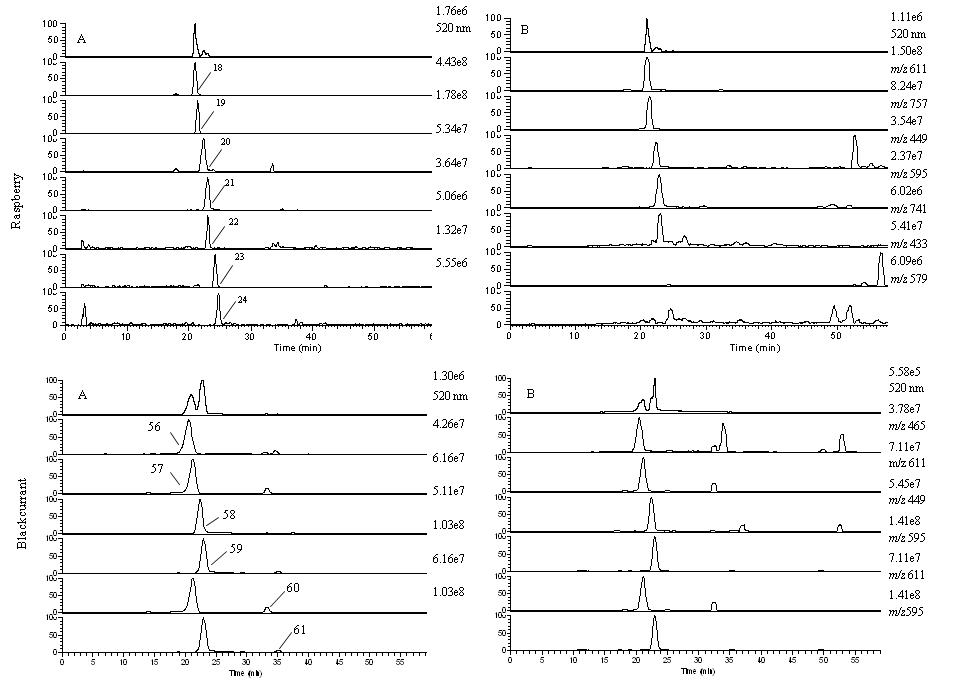
**

**Figure S1: LC-MS traces for raspberry and blackcurrant extracts (A) and IVD extracts (B).** For each panel the top trace represents the scan at 520 nm. The masses for each peak are given along with the full scale deflection value. Peak labels correspond with the putative identities in Table S1.
