# Supplementary material for: Persistence of Anticancer Activity in Berry Extracts after Simulated Gastrointestinal Digestion and Colonic Fermentation
Source: PLoS One. 2012 Nov 21;7(11):e49740. doi: 10.1371/journal.pone.0049740 (PMC3504104; doi:10.1371/journal.pone.0049740)
Supplement: Table S1 — Total phenol and anthocyanin content of berry extracts pre-simulated digestion, IVD and post SPE, n = 8. (DOCX) [file pone.0049740.s002.docx]

**Table S1.** Total phenol and anthocyanin content of berry extracts pre-simulated digestion, IVD and post SPE, n = 8.

|  | **Total Phenol Content**  **(µg/mL GAE)** | | | **Total Anthocyanin Content**  **(µg/mL CGE)** | | |
| --- | --- | --- | --- | --- | --- | --- |
| **Berry** | **Pre-digest** | **IVD** | **Post-SPE** | **Pre-digest** | **IVD** | **Post-SPE** |
| Raspberry | 1804 ± 22 | 255 ± 36 | 192 ± 26 | 818 ± 23 | 186 ± 19 | 71 ± 22 |
| Strawberry | 3109 ± 44 | 503 ± 22 | 392 ± 31 | 580 ± 32 | 169 ± 29 | 80 ± 14 |
| Blackcurrant | 12091 ± 54 | 772 ± 41 | 329 ± 43 | 8824 ± 41 | 159 ± 33 | 134 ± 26 |
