# Supplementary material for: Persistence of Anticancer Activity in Berry Extracts after Simulated Gastrointestinal Digestion and Colonic Fermentation
Source: PLoS One. 2012 Nov 21;7(11):e49740. doi: 10.1371/journal.pone.0049740 (PMC3504104; doi:10.1371/journal.pone.0049740)
Supplement: Table S2 — Putative identities and quantities of (poly)phenolic compounds detected in berry extracts before and after IVDa. (DOCX) [file pone.0049740.s003.docx]

Table S2: Putative identities and quantities of (poly)phenolic compounds detected in berry extracts before and after IVD.^a^

| **Peak Number^b^** | | **Putative Identity** | **Pre-digest**  **(μg/mL)** | **After IVD**  **(μg/mL)** | | **Recovery (%)^c^** |
| --- | --- | --- | --- | --- | --- | --- |
| **Raspberry** | | | | | | |
| 1 | | Sanguiin H-6 | 181 ± 8 | 96 ± 5 | | 54 ± 4 |
| 2 | | Lambertianin C | 151 ± 3 | 29 ± 3 | | 19 ± 0.0* |
| 3 | | Sanguiin H-10 isomer | 20 ± 3 | 19 ± 2 | | 99 ± 14 |
| 4 | | *p*-Coumaroyl-*O*-hexoside | 6.1 ± 0.5 | 8.1 ± 0.3 | | 133 ± 8 |
| 5 | | Feruloyl-*O*-hexoside | 3.9 ± 0.5 | 3.8 ± 0.3 | | 94 ± 13 |
| 6 | | Sanguiin H-10 isomer | 3.2 ± 0.2 | 6.3 ± 0.1 | | 199 ± 6 |
| 7 | | Ellagic acid-*O*-pentoside | 3.1 ± 0.2 | 5.2 ± 0.2 | | 171 ± 6 |
| 8 | | Ellagic acid-*O*-pentoside | 2.3 ± 0.1 | 3.1 ± 0.1 | | 133 ± 4 |
| 9 | | Ellagic acid derivative | 2.8 ± 0.2 | 4.9 ± 0.1 | | 173 ± 7 |
| 10 | | Kaempferol-*O*-hexoside | 3.1 ± 0.1 | 9.0 ± 0.3 | | 291 ± 3 |
| 11 | | Quercetin-*O*-glucuronide | 2.0 ± 0.1 | 3.6 ± 0.2 | | 181 ± 5 |
| 12 | | Ellagic acid derivative | 1.1 ± 0.1 | 1.5 ± 0.1 | | 136 ± 9 |
| 13 | | Ellagic acid-*O*-pentoside | 1.5 ± 0.1 | 0.9 ± 0.1 | | 63 ± 7 |
| 14 | | Hydroxycinnamate derivative | 0.9 ± 0.1 | 0.8 ± 0.1 | | 100 ± 11 |
| 15 | | Unknown | 0.6 ± 0.0* | 1.1 ± 0.0 | | 168 ± 8 |
| 16 | | Unknown | 0.5 ± 0.0 | 0.6 ± 0.0 | | 116 ± 8 |
| 17 | | Unknown | 0.3 ± 0.0 | 0.6 ± 0.0 | | 178 ± 2 |
| 18 | | Cyanidin-3-*O*-sophoroside | 125 ± 3 | 56 ± 3 | | 31 ± 3 |
| 19 | | Cyanidin-3-*O*-glucosylrutinoside | 43 ± 1 | 32 ± 1 | | 78 ± 3 |
| 20 | | Cyanidin-3-*O*-glucoside | 26 ± 0.0 | 13 ± 0 | | 47 ± 1 |
| 21 | | Cyanidin-3-*O*-rutinoside & pelargonidin-3-*O*-sophoroside | 17 ± 1 | 12 ± 0.0 | | 69 ± 3 |
| 22 | | Pelargonidin-3-*O*-glucosylrutinoside | 2.0 ± 0.0 | 1.9 ± 0.0 | | 94 ± 3 |
| 23 | | Pelargonidin-3-*O*-glucoside | 3.3 ± 0.1 | 0.8 ± 0.1 | | 29 ± 3 |
| 24 | | Pelargonidin-3-*O*-rutinoside | 1.4 ± 0.2 | 1.2 ± 0.1 | | 79 ± 14 |
| **Strawberry** | | | | | | |
| 25 | Proanthocyanidin B-type trimer | | 4.6 ± 0.1 | n.d. | | n.d. |
| 26 | Catechin | | 23 ± 1 | 12 ± 1 | | 51 ± 6 |
| 27 | bis-Hexahydroxydiphenoyl-*O*-glucoside | | 21 ± 1 | 20 ± 2 | | 94 ± 7 |
| 28 | Ellagitannin derivative-1 | | 33 ± 2 | 49 ± 2 | | 159 ± 7 |
| 29 | Epicatechin | | 11 ± 1 | 7.5 ± 0.5 | | 66 ± 5 |
| 30 | *p*-Coumaroyl-*O*-hexoside | | 17 ± 1 | 14 ± 0 | | 72 ± 8 |
| 31 | Sanguiin H-6 | | 56 ± 2 | 49 ± 2 | | 90 ± 3 |
| 32 | Galloyl bis-hexahydroxydiphenoyl-*O*-glucoside | | 54 ± 0 | 30 ± 0 | | 56 ± 6 |
| 33 | Ellagitannin derivative-2 | | 6.2 ± 0.6 | 22 ± 1 | | 362 ± 10 |
| 34 | Catechin-*O*-hexoside | | 5.1 ± 0.2 | 3.7 ± 0.3 | | 75 ± 6 |
| 35 | Ellagic acid-*O*-glucuronide | | 1.1 ± 0.1 | 3.4 ± 0.2 | | 296 ± 9 |
| 36 | Ellagic acid derivative | | 3.6 ± 0.3 | 11 ± 1 | | 294 ± 8 |
| 37 | Feruloyl-*O*-hexoside | | 2.4 ± 0.2 | 17 ± 1 | | 677 ± 8 |
| 38 | Quercetin-*O*-glucuronide | | 21 ± 0 | 65 ± 2 | | 300± 7 |
| 39 | Kaempferol-3-*O*-glucoside | | 1.8 ± 0.1 | 5.7 ± 0.3 | | 305 ± 5 |
| 40 | Kaempferol-*O*-acetylhexoside | | 4.0 ± 0.2 | 12 ± 1 | | 304 ± 4 |
| 41 | Kaemferol-3-*O*-malonylglucoside | | 4.0 ± 0.0 | 13 ± 1 | | 311 ± 14 |
| 42 | Hydroxycinnamate derivative | | 0.6 ± 0.1 | 0.3 ± 0.1 | | 54 ± 17 |
| 43 | Pelargonidin-3-*O*-glucoside | | 21± 1 | 11 ± 1 | | 52 ± 6 |
| 44 | Pelargonidin-3-*O*-malonylglucoside | | 5.2 ± 0.7 | 3.6 ± 0.3 | | 74 ± 13 |
| **Blackcurrant** | | | | | | |
| 45 | | 5-*O*-Caffeoylquinic acid | 9.4 ± 0.8 | | 15 ± 1 | 165 ± 9 |
| 46 | | Caffeoyl-*O*-glucoside | 3.8 ± 0.2 | | 2.9 ± 0.3 | 76 ± 8 |
| 47 | | Myricetin-3-*O*-rutinoside | 7.9 ± 0.8 | | 9.4 ± 1.0 | 120 ± 11 |
| 48 | | Myricetin-3-*O*-galactoside | 6.2 ± 0.2 | | 5.5 ± 0.1 | 89± 3 |
| 49 | | Myricetin-3-*O*-glucoside | 24 ± 2 | | 24 ± 2 | 97 ± 7 |
| 50 | | Quercetin-3-*O*-rutinoside | 17 ± 1 | | 22 ± 1 | 129 ± 6 |
| 51 | | Quercetin-3-*O*-galactoside | 6.1 ± 0.6 | | 6.2 ± 0.4 | 102 ± 10 |
| 52 | | Quercetin-3-*O*-glucoside | 29 ± 2 | | 32 ± 1 | 112 ± 5 |
| 53 | | Quercetin-*O*-malonylhexoside | 3.6 ± 0.2 | | 5.3 ± 0.4 | 138 ± 8 |
| 54 | | Kaempferol-*O*-hexoside | 7.6 ± 0.5 | | 9.6 ± 0.7 | 135 ± 7 |
| 55 | | Isorhamnetin-*O*-malonylhexoside | 4.6 ± 0.2 | | 4.3 ± 0.3 | 94 ± 4 |
| 56 | | Delphinidin-3-*O*-glucoside | 95 ± 6 | | 24 ± 2 | 25 ± 7 |
| 57 | | Delphinidin-3-*O*-rutinoside | 117 ± 7 | | 42 ± 3 | 36 ± 6 |
| 58 | | Cyanidin-3-*O*-glucoside | 88 ± 3 | | 29 ± 1 | 33 ± 4 |
| 59 | | Cyanidin-3-*O*-rutinoside | 155 ± 2 | | 72 ± 4 | 44 ± 3 |
| 60 | | Cyanidin-3-*O*-coumaroylglucoside | 7.3 ± 0.7 | | 3.0 ± 0.1 | 39 ± 10 |
| 61 | | Delphinidin-3-*O*-coumaroylglucoside | 6.2 ± 0.5 | | 0.5 ± 0.0 | 8 ± 4 |

^a^Full details of putative peak identities are presented in Table S1 (see supplementary information).

^b^For peak numbers see Table S1.

^c^% recoveries of compounds after IVD as a percentage of the amounts present prior to IVD ± standard deviation (n = 3).
