# Supplementary material for: Persistence of Anticancer Activity in Berry Extracts after Simulated Gastrointestinal Digestion and Colonic Fermentation
Source: PLoS One. 2012 Nov 21;7(11):e49740. doi: 10.1371/journal.pone.0049740 (PMC3504104; doi:10.1371/journal.pone.0049740)
Supplement: Table S3 — Putative identities of peaks detected in pre-digested berry extracts. (DOCX) [file pone.0049740.s004.docx]

**Table S3:** Putative identities of peaks detected in pre-digested berry extracts

| **Peak no.** | **Rt (min)** | | **[M+H] *(m/z)*** | **MS^2^ *(m/z)*** | **λmax**  **(nm)** | **Putative Identity** |
| --- | --- | --- | --- | --- | --- | --- |
| **Raspberry** | | | | | | |
| 1 | 29.01 | 1887 (1869) | | multiple | 280 | Sanguiin H-6 |
| 2 | 28.02 | 1422 (1401) | | multiple | 280 | Lambertinian C |
| 3 | 25.56 | 1585 (1567) | | multiple | 280 | Sanguiin H-10 isomer |
| 4 | 23.55 | (371, 325) | | 163 | 320 | *p*-Coumaroyl-*O*-hexoside |
| 5 | 25.05 | (355) | | 193 | 330 | Feruloyl-*O*-hexoside |
| 6 | 26.95 | (1567) | | multiple | 285 | Sanguiin H-10 isomer |
| 7 | 28.73 | (433) | | 301 | 360 | Ellagic acid-*O*-pentoside |
| 8 | 30.87 | (433) | | 301 | 365 | Ellagic acid-*O*-pentoside |
| 9 | 31.83 | nd | | nd | 360 | Ellagic acid derivative |
| 10 | 33.57 | 449 | | 287 | 355 | Kaempferol-*O*-hexoside |
| 11 | 34.05 | 479 | | 303 | 355 | Quercetin-*O*-glucuronide |
| 12 | 35.98 | (447) | | 301 | 350 | Ellagic acid-*O*-rhamnoside |
| 13 | 36.44 | (475) | | nd | 360 | Ellagic acid derivative |
| 14 | 37.18 | nd | | nd | 310 | Hydroxycinnamate derivative |
| 15 | 38.15 | nd | | nd | 350 | Unknown |
| 16 | 41.65 | nd | | nd | 370 | Unknown |
| 17 | 43.1 | nd | | nd | 330-370 | Unknown |
| 18 | 20.97 | 611 | | 287 | 515 | Cyanidin-3-*O*-sophoroside |
| 19 | 21.37 | 757 | | 287 | 515 | Cyanidin-3-*O*-glucosylrutinoside |
| 20 | 22.35 | 449 | | 287 | 515 | Cyanidin-3-*O*-glucoside |
| 21 | 22.99 | 595/595 | | 287, 271 | 520 | Cyanidin-3-*O*-rutinoside & pelargonidin-3-*O*-sophoroside |
| 22 | 23.05 | 741 | | 287 | 500 | Pelargonidin-3-*O*-glucosylrutinoside |
| 23 | 24.19 | 433 | | 287 | 500 | Pelargonidin-3-*O*-glucoside |
| 24 | 24.75 | 579 | | 271 | 500 | Pelargonidin-3-*O*-rutinoside |
| **Strawberry** | | | | | | |
| 25 | 14.06 | | 867 | 579 | 275 | Proanthocyanidin B-type trimer |
| 26 | 15.47 | | 291 | 207 | 285 | Catechin |
| 27 | 18.23 | | (783) | 481, 301 | 280 | bis-Hexahydroxydiphenoyl-*O*-glucoside |
| 28 | 18.66 | | nd | nd | 280 | Ellagitannin derivative-1 |
| 29 | 22.94 | | 291 | 225 | 285 | Epicatechin |
| 30 | 23.36 | | (325) | 163 | 315 | *p*-Coumaroyl-*O*-hexoside |
| 31 | 31.47 | | 1887 (1869) | multiple | 280 | Sanguiin H-6 |
| 32 | 34.99 | | (935) | multiple | 280 | Galloyl bis-hexahydroxydiphenoyl-*O*-glucoside |
| 33 | 49.67 | | nd | nd | 275 | Ellagitannin derivative-2 |
| 34 | 27.85 | | 451 | 289 | 280-300 | Catechin-*O*-hexoside |
| 35 | 30.84 | | 479 | 303 | 370 | Ellagic acid-*O*-glucuronide |
| 36 | 31.06 | | nd | nd | 370 | Ellagic acid derivative |
| 37 | 33.52 | | (355) | nd | 330 | Feruloyl-*O*-hexoside |
| 38 | 34.03 | | 479 | 303 | 360 | Quercetin-3-*O*-glucuronide |
| 39 | 36.86 | | 449 | 287 | 360 | Kaempferol-3-*O*-glucoside |
| 40 | 37.19 | | 463 | 287 | 360 | Kaempferol-3-*O*-glucuronide |
| 41 | 39.28 | | 535 | 287 | 360 | Kaemferol-3-*O*-malonylglucoside |
| 42 | 46.39 | | nd | nd | 320 | Hydroxycinnamate derivative |
| 43 | 24.18 | | 433 | 271 | 495 | Pelargonidin-*3-O-*glucoside |
| 44 | 28.92 | | 519 | 271 | 495 | Pelargonidin-3-*O*-malonylglucoside |
| **Blackcurrant** | | | | | | |
| 45 | 19.97 | | 355 | 163 | 330 | 5-*O*-Caffeoylquinic acid |
| 46 | 24.31 | | (341) | 179 | 330 | Caffeoyl-*O*-glucoside |
| 47 | 29.78 | | 627 | 319 | 360 | Myricetin-3-*O*-rutinoside |
| 48 | 30.50 | | 481 | 319 | 360 | Myricetin-3-*O*-galactoside |
| 49 | 30.81 | | 481 | 319 | 360 | Myricetin-3-*O*-glucoside |
| 50 | 32.80 | | 611 | 303 | 355 | Quercetin-3-*O*-rutinoside |
| 51 | 34.06 | | 465 | 303 | 355 | Quercetin-3-*O*-galactoside |
| 52 | 34.38 | | 465 | 303 | 355 | Quercetin-3-*O*-glucoside |
| 53 | 36.46 | | 551 | 303 | 355 | Quercetin-*O*-malonylhexoside |
| 54 | 37.25 | | 449 | 287 | 355 | Kaempferol-*O*-hexoside |
| 55 | 39.47 | | 565 | 317 | 350 | Isorhamnetin-*O*-malonylhexoside |
| 56 | 33.59 | | 465 | 303 | 525 | Delphinidin-3-*O*-glucoside |
| 57 | 35.37 | | 611 | 303 | 525 | Delphinidin-3-*O*-rutinoside |
| 58 | 20.90 | | 449 | 303 | 515 | Cyanidin-3-*O*-glucoside |
| 59 | 21.30 | | 595 | 303 | 515 | Cyanidin-3-*O*-rutinoside |
| 60 | 22.83 | | 611 | 303 | 530 | Delphinidin-3-*O*-coumaroylglucoside |
| 61 | 23.34 | | 595 | 303 | 520 | Cyanidin-3-*O*-coumaroylglucoside |

nd = not determined, Previous work and available literature was used to putatively identify compounds in the samples [1-4]. Putative identifications are backed by negative mode MS work and [M-H] values are given in parentheses.

**References**

1. Aaby, K., Ekeberg, D., Skrede, G. Characterization of phenolic compounds in strawberry (*Fragaria* x *ananassa*) fruits by different HPLC detectors and contribution of individual compounds to total antioxidant capacity. *J. Agric. Food Chem.* 2007, *55,* 4395–4406.

2. Maatta-Riihinen, K. R., Kamal-Eldin, A., Torronen, A. R. Identification and quantification of phenolic compounds in berries of *Fragaria* and *Rubus* species (Family rosaceae). *J. Agric. Food Chem.* 2004, *52,* 6178–6187.

3. McDougall, G. J., Dobson, P., Smith, P., Blake, A., Stewart, D. Assessing potential bioavailability of raspberry anthocyanins using an in vitro digestion system. *J. Agric. Food Chem.* 2005, *53,* 5896–5904.

4. McDougall, G. J., Dobson, P., Shpiro, F., Smith, P., Stewart, D., Fyffe, S. Assessing bioavailability of soft fruit polyphenols *in vitro.* *Acta Hort.* 2007, *744,* 135–
